# Authors’ response: Chikungunya infection in travellers to Thailand: additional United Kingdom cases identified by specialist laboratory

**DOI:** 10.2807/1560-7917.ES.2019.24.24.1900367

**Published:** 2019-06-13

**Authors:** Emilie Javelle, Davidson H Hamer, Vanessa Field, Shilan Jmor, Philippe Gautret

**Affiliations:** 1 Laveran Military Teaching Hospital, Marseille, France; 2 IHU-Méditerranée Infection, Marseille, France; 3 Aix Marseille Univ, IRD, AP-HM, SSA, VITROME, Marseille, France; 4 Department of Global Health, Boston University School of Public Health, Boston, United States; 5 Section of Infectious Diseases, Department of Medicine, Boston Medical Center, Boston, United States; 6 Chair of the Tracking and Communications Working Group, GeoSentinel, London, United Kingdom; 7 Hospital for Tropical Diseases, London, United Kingdom

**Keywords:** chikungunya, Thailand, travel, surveillance network

**To the editor:** We appreciate the thoughtful response of our colleagues at the chikungunya reference laboratory at the National Infection Service, Public Health England in regard to our rapid communication on travellers affected by the chikungunya outbreak in Thailand [1]. We read with interest their description of two additional travellers from the United Kingdom (UK) who were infected with chikungunya in the same outbreak. There have been several other patients who acquired chikungunya in southern Thailand in the first 4 months of 2019, including five Finnish travellers [2], and an additional seven confirmed cases have been reported within the GeoSentinel/EurotravNet network since the publication of our report (all of these cases occurred within a similar time frame, from mid-January to late March 2019). Overall, including those described in our original article, 23 cases of chikungunya acquired by international travellers in Thailand in late 2018 and early 2019 have been reported in the literature (PubMed database), all in European travellers except for two in Israeli travellers. These numbers are likely to be an underestimation of the morbidity of this outbreak among travellers, given the disease incidence in the local population. Currently, the number of cases reported locally by week is declining. In 2019, as at 13 May, Thailand has reported 3,379 chikungunya cases in 23 provinces, with no deaths [3]. This is around twice the number of cases that were registered at the time of our original publication (1,652 patients as at 10 February). The most affected provinces are located in the southern part of the country.

GeoSentinel is a surveillance network that uses returning travellers and migrants as sentinels of disease outbreaks. We appreciate that there are recognised limitations to this approach, including the lack of a denominator, which means that incidence rates in returning travellers cannot be calculated. We also acknowledge that there may be incomplete reporting, because returning travellers are also seen in institutions that do not belong to the network in their respective countries [4]. However, the strength of this approach is that visited places, respective travel dates and clinical features are systematically collected by the network sites.

We agree with our colleagues’ recognition of the importance of laboratory networks for detecting infectious disease outbreaks, as well as the need for coordinated communication regarding diseases of public health importance to health officials at the country, regional and global levels. We also concur that a laboratory-based system of routine notifications of imported infections—in particular, arboviral infections caused by chikungunya, dengue, yellow fever and Zika viruses—with centralised reporting would be useful for helping to identify and track outbreaks of infectious diseases in novel locations. These data would be an excellent complement to clinical provider networks such as GeoSentinel/Eurotravnet.
